# Why PLoS Became a Publisher

**DOI:** 10.1371/journal.pbio.0000036

**Published:** 2003-10-13

**Authors:** Patrick O Brown, Michael B Eisen, Harold E Varmus

## Abstract

Public Library of Science has grown from a grassroots movement to a nonprofit publisher, in order to catalyze change towards open-access publishing of the scientific literature

Communication among scientists has undergone a revolution in the last decade, with the movement of scientific publication to a digital medium and the emergence of the Internet as the primary means for distributing information. Millions of articles are, in principle, just a mouse click away from our computers. For many of us, PDFs have replaced printed journals as the primary form in which we read about the work of our colleagues.

Yet we have barely begun to realize the potential of this technological change. For practicing scientists, it provides myriad opportunities to expand and improve the ways we can use the scientific literature. Equally important, it is now possible to make our treasury of scientific information available to a much wider audience, including millions of students, teachers, physicians, scientists, and other potential readers, who do not have access to a research library that can afford to pay for journal subscriptions.

We founded the Public Library of Science three years ago to work toward realizing these opportunities. We began as a grassroots organization of scientists, advocating the establishment and growth of online public libraries of science, such as the National Institutes of Health's PubMed Central, to provide free and unrestricted access to the scientific literature. Today, with the launch of *PLoS Biology*, we take on a new role as publishers, to demonstrate that high-quality journals can flourish without charging for access.

## Open Access

*PLoS Biology*, and every PLoS journal to follow, will be an open-access publication–everything we publish will immediately be freely available to anyone, anywhere, to download, print, distribute, read, and use without charge or other restrictions, as long as proper attribution of authorship is maintained. Our open-access journals will retain all of the qualities we value in scientific journals—high standards of quality and integrity, rigorous and fair peer-review, expert editorial oversight, high production standards, a distinctive identity, and independence. Although most readers will be satisfied with the free and unrestricted use of the online edition (including the right to print their own copies), a printed edition of *PLoS Biology* will be made available, for the cost of printing and distribution, to readers who prefer the convenience and browseability of the traditional paper format. And the full contents of every issue will immediately be placed in the National Library of Medicine's public online archive, PubMed Central, guaranteeing their permanent preservation and free accessibility.

Our aim is to catalyze a revolution in scientific publishing by providing a compelling demonstration of the value and feasibility of open-access publication. If we succeed, everyone who has access to a computer and an Internet connection will be a keystroke away from our living treasury of scientific and medical knowledge. This online public library of science will form a valuable resource for science education, lead to more informed healthcare decisions by doctors and patients, level the playing field for scientists in smaller or less wealthy institutions, and ensure that no one will be unable to read an important paper just because his or her institution does not subscribe to a particular journal.

Open access will also enable scientists to begin transforming the scientific literature into something far more useful than the electronic equivalent of millions of individual articles in rows of journals on library shelves. The ability to search, in an instant, an entire scientific library for particular terms or concepts, for methods, data, and images—and instantly retrieve the results—is only the beginning. Freeing the information in the scientific literature from the fixed sequence of pages and the arbitrary boundaries drawn by journals or publishers— the electronic vestiges of paper publication—opens up myriad new possibilities for navigating, integrating, “mining,” annotating, and mapping connections in the high-dimensional space of scientific knowledge.

Consider how the open availability and freedom to use the complete archive of published DNA sequences in the GenBank, EMBL, and DDBJ databases inspired and enabled scientists to transform a collection of individual sequences into something incomparably richer. With great foresight, it was decided in the early 1980s that published DNA sequences should be deposited in a central repository, in a common format, where they could be freely accessed and used by anyone. Simply giving scientists free and unrestricted access to the raw sequences led them to develop the powerful methods, tools, and resources that have made the whole much greater than the sum of the individual sequences. Just one of the resulting software tools—BLAST—performs 500 trillion sequence comparisons annually! Imagine how impoverished biology and medicine would be today if published DNA sequences were treated like virtually every other kind of research publication—with no comprehensive database searches and no ability to freely download, reorganize, and reanalyze sequences. Now imagine the possibilities if the same creative explosion that was fueled by open access to DNA sequences were to occur for the much larger body of published scientific results.

## Paying the Bill for Open Access

The benefits of open access are incontestable. The questions and concerns that remain focus on finances. As everyone acknowledges, publishing a scientific journal costs money—the more rigorous the peer review, the more efficient and expert the editorial oversight, the more added features and the higher the production standards, the greater the cost to publishers. Most journals today depend on subscriptions and site-licensing fees for most of their revenue. Since these access tolls are incompatible with open access, how will newly formed open-access journals pay their bills, and how will the traditional journals that have served the scientific community for many years survive in an open-access world?

Because publishing is an integral part of the research process, a natural alternative to the subscription model is to consider the significant but relatively small costs of open-access publication as one of the fundamental costs of doing research. The institutions that sponsor research intend for the results to be made available to the scientific community and the public. If these research sponsors also paid the essential costs of publication—amounting, by most estimates, to less than 1% of the total spent on sponsored research (statistics found at http://dx.doi.org/10.1371/journal.pbio.0000036.sd001)—we would retain a robust and competitive publishing industry and gain the benefit of universal open access.

The subscription model—in which the publishers own the works they publish and dictate the conditions under which they can be accessed or used—is sometimes presented as the only possible way to pay for scientific publishing. This pay-for-access model was well suited to a world in which the most efficient way to record and transmit scientific information on a large scale was by printing and distributing scientific journals. When each incremental copy represented a significant expense to the publisher, any sustainable business model depended on recovering the cost for each copy—the recipients of the information had to pay for access. But the essential rationale of the pay-for-access model has disappeared, now that electronic publication and Internet distribution have become routine. Instead, this business model is what stands in the way of all the benefits of open access.

Asking research sponsors to pay for publication of the research they support may seem to impose new financial burdens on the government agencies, foundations, universities, and companies that sponsor research. But these organizations already pay most of the costs of scientific publishing—a huge fraction of the US$9 billion annual revenue of scientific, medical, and technology journals comes from subscriptions, site licenses, and publication fees ultimately billed to grants or employers. Much of the rest is borne by society in the form of increments to university tuitions; healthcare costs, including drug prices; and state and federal taxes that subsidize healthcare, libraries, and education. Surely the cost of open-access digital publishing cannot, in total, be more than we are already paying under the subscription and licensing model. By simply changing the way we support the scientific publishing enterprise, the scientific community and public would preserve everything we value in scientific publishing and gain all of the benefits of open access.

There are reasons to believe that open-access publishing would cost significantly less than the current system. Today, each journal has a monopoly on a resource vital to scientists—the unique collection of articles it has published. Anyone who depends on the information in a specific article has no choice but to pay whatever price the publisher asks (or find a colleague or library that has done so). Because scientists are so dependent on ready access to previously published work, publishers are able to set their prices with little fear of subscription cancellations. Indeed, journal prices have been rising at a rate well in excess of inflation, straining the budgets of universities, hospitals, and research institutions. Open access would eliminate monopolies over essential published results, diminishing profit margins and creating a more efficient market for scientific publishing—a market in which publishers would compete to provide the best value to authors (high quality, selectivity, prestige, a large and appreciative readership) at the best price.

## Joining Forces

In recent months, we have witnessed a remarkable surge of awareness and support for open-access publication, both within the scientific community and in the public at large, exemplified by recent newspaper articles and editorials supporting PLoS and open access; by the recent introduction of the Public Access to Science Act in the United States Congress; by the Bethesda Workshop on Open Access; and by public statements of support from organizations as diverse as the NIH Council of Public Representatives, the Association of Research Libraries, and the Susan G. Komen Breast Cancer Foundation. Achieving universal open access will require action from funding agencies and institutions.

The Howard Hughes Medical Institute, the largest private sponsor of biomedical research in the United States, has already taken a leading role in promoting open access. They will provide each of their investigators with supplemental funds to cover the costs of publishing in open-access journals like *PLoS Biology.* Other major institutional sponsors of biomedical research are actively considering similar policies.

Private foundations with a commitment to science and education have contributed generously to this cause. Like any new business, PLoS needed to raise funds to cover our startup costs. A generous grant from the Gordon and Betty Moore Foundation enabled PLoS to launch our nonprofit publishing venture. Other individuals and organizations, notably the Irving A. Hansen Foundation, also provided generous and welcome support. These start-up funds made it possible for us to assemble an outstanding editorial board and staff, who have today accomplished the extraordinary feat of launching a new publisher and a premiere journal from scratch in less than nine months.

The opposition of most established journals to open access has left it to new journals like *PLoS Biology* and BioMed Central's *Journal of Biology* to lead the way. These new journals face a double challenge. First, we are introducing an unfamiliar model—open-access publication. Second, any new journal, even one with the stringent standards and the extraordinary editorial team of *PLoS Biology*, must begin without the established “brand name” of the older journals, which, like a designer logo, elevates the perceived status of the articles that bear it. With all that is at stake in the choice of a journal in which to publish—career advancement, grant support, attracting good students and fellows—scientists who believe in the principle of open access and wish to support it are confronted with a difficult dilemma. We applaud the courage and pioneering spirit of the authors who have chosen to send to a fledgling journal the outstanding articles you will read in the premiere issues of *PLoS Biology.* In the end, it's the contributions of these authors that will make *PLoS Biology* a success.

