# Correction: Why PLoS Became a Publisher

**DOI:** 10.1371/journal.pbio.3003714

**Published:** 2026-03-17

**Authors:** PLOS Biology

The link to the statistics in the section “Paying the Bill for Open Access” paragraph two is broken. A copy of the file can be viewed below.

## Supporting information

S1 File Statistical data. (DOC)
