# Supplementary material for: Correction: Why PLoS Became a Publisher
Source: PLoS Biol. 2026 Mar 17;24(3):e3003714. doi: 10.1371/journal.pbio.3003714 (PMC12994835; doi:10.1371/journal.pbio.3003714)
Supplement: S1 File — (DOC) [file pbio.3003714.s001.doc]

**Note: This analysis is based on data extracted from the 490,542 Medline records for articles published in 2002, kindly provided by Sheldon Kotzin (E-mail: KOTZINS@mail.nlm.nih.gov).**

Data:

Total 2002 citations in Medline = 490,542

Support, US government, Public Health Service (PHS) = 58,521 (12%)

(in a study done in January 2003, the National Institutes of Health [NIH] accounted for 87% of the PHS support)

Support, US government, non-PHS = 18,728 (4%)

Support, non-US government = 169,282 (35%) (includes foreign grants and grants from US pharmaceutical and medical device companies)

Analysis: We consider two estimates of average investment per publication resulting from NIH-sponsored research. The first estimate probably greatly overestimates the number of articles resulting from NIH-sponsored research. The second estimate probably slightly underestimates this number, but we believe it to be the more accurate estimate:

1. If we suppose that the Medline-indexed articles that did not acknowledge a research sponsor were in fact sponsored research and that the distribution of sponsorship paralleled that of the articles that did cite one or more research sponsors, we can estimate that the fraction of NIH supported research is 12 × 0.87% (of 490,542 articles). We assume that although many of those articles are probably jointly funded, the distribution of sponsorship in those cases is reflected in the fraction of all acknowledged support that comes from NIH. This leads to an estimated 101,305 articles in 2002 resulting from NIH-sponsored research. Let’s assume that the research reported in those articles was all funded in the year 2000. The NIH budget in 2000 was US$18 billion. This comes to US$177,681 NIH support per published paper. At a publication cost of US$1,500 per published paper, the average cost of publication would be 0.84% of the cost of the funded research.

2. If we assume that every article reporting NIH-funded research included an acknowledgment of that support (i.e., the articles that do not cite any support are funded by the authors’ institutional or corporate employers or are not primary research articles – e.g., case reports, commentary, teaching articles, reviews, etc.). Then the total comes to 50,913 (58,521 × 0.87) articles reporting NIH-supported research, for an average NIH investment of US$354,000 per published article. By this estimate, at US$1,500 per published paper, the cost of publication would be just 0.42% of the cost of the research being reported.
